# Response of soil organic carbon and soil aggregate stability to changes in land use patterns on the Loess Plateau

**DOI:** 10.1038/s41598-024-82300-2

**Published:** 2024-12-30

**Authors:** Zhandong Pan, Xuemei Cai, Yongming Bo, Changsheng Guan, Liqun Cai, Fasih Ullah Haider, Xuchun Li, Haixia Yu

**Affiliations:** 1 College of Forestry, Gansu Agricultural University, Lanzhou, 730070 China; 2 State Key Laboratory of Aridland Crop Science, Gansu Agricultural University, Lanzhou, 730070 China; 3 Dingxi Institute of Soil and Water Conservation Science Research, Dingxi, 743000 China; 4 College of Resources and Environment Sciences, Gansu Agricultural University, Lanzhou, 730070 China

**Keywords:** Soil aggregate stability, Soil organic carbon, Loess Plateau, Returned to forestland and grassland, Land use patterns, Ecology, Environmental sciences

## Abstract

Land use change can significantly alter the proportion of soil aggregates, thereby influencing aggregate stability and distribution of soil organic carbon (SOC). However, there is minimal research on the variations in the distribution of soil aggregates, aggregate stability, and SOC in soil aggregates following land use change from farmland (FL) to forest and grassland in the Loess Plateau region of China. Select six land use patterns (farmland (FL), abandoned cropland (ACL), *Medicago sativa* (MS), natural grassland (NG), *Picea asperata* Mast*.* (PA), *Platycladus orientalis* (L.) Franco (PO)) on the Loess Plateau in China and collect undisturbed soil samples. These six land use patterns have similar geographical characteristics. The distribution of aggregates and the aggregate-associated SOC contents under the six land use patterns were measured at the 0–10 cm, 10–30 cm and 30–50 cm depths. The results showed that forestland and grasslands converted from FL significantly increased the aggregates (> 5 mm) content, mean weight diameter (MWD), and geometric mean diameter (GMD) but decreased the aggregates (< 0.25 mm) content. Compared with FL, the values at the 0–50 cm depth under PA, NG, MS, PO and ACL increased by 473.71–732.55%, 283.98–724.60%, 179.06–634.12%, 142.31–413.50% and 110.25–213.34%, respectively, for MWD and by 244.04–607.77%, 141.68–666.67%, 52.39–483.33%, 50.49–214.43%, and 35.23–64.29%, respectively, for GMD. Land use patterns and soil aggregate size had obvious influences on SOC content, SOC content in soil and aggregates decreased under ACL. In other forestland and grasslands, The SOC content in bulk soil, > 5 mm, 2–5 mm, 1–2 mm, 0.5–1 mm, 0.25–0.5 mm, and < 0.25 mm aggregates at the 0–50 cm depth after afforestation increased by 20.75–125.87%, 14.50–163.64%, − 11.86–118.18%, 9.65–150.95%, 38.28–126.49%, 51.26–165.87% and − 15.59–163.37%, respectively, Compared to FL. The contributions of different aggregates particle sizes to the increase in SOC content in bulk soil were 104.74%, 7.86%, 4.76%, 6.23%, 5.37%, and − 21.97%, respectively. MWD and GMD were positively correlated with SOC content in aggregates (1 mm), SOC content in bulk soil and aggregates. Although SOC content in bulk soil and different aggregates particle sizes under NG and PA were significantly higher that than under MS and PO, the soil macroaggregate content, MWD, and GMD under PO and NG were higher than that under PA and MS. These findings suggest that converted FL into PO and NG significantly improved soil structure and also increased SOC content. Therefore, in the process of transforming land use patterns on the Loess Plateau, the proportion of forest land and grassland should be appropriately increased to improve soil carbon storage and quality. The results of this study provides a theoretical basis and scientific basis for the scientific evaluation and understanding of soil organic carbon accumulation and distribution under different land use patterns in the Loess Plateau region of China.

## Introduction

Land use change is a crucial factor contributing to global climate perturbations, holding significant relevance for biogeochemical cycles on regional and global levels^1–3^. The change in land use directly affects SOC’s content and distribution and indirectly affects SOC by influencing factors related to its formation and transformation^4^. The most important reason for global SOC loss was changes in land use patterns and management practices, followed by changes in environmental factors such as rising temperatures and precipitation^5,6^. Reasonable land use change management can promote the accumulation of SOC and play a key role in mitigating global climate change^7,8^. SOC stock and soil structure are often used as key indicators of the extent of soil degradation or improvement resulting from land use change^9–11^. Soil aggregates as an important indicator of soil quality, are closely related to soil bulk density, moisture, porosity, etc.^12^. A good soil structure mainly depends on the soil aggregates stability and distribution, and also can improve soil production, increasing soil erosion resistance, and carbon sequestration^13^. Therefore, exploring the distribution of soil aggregate structure and SOC sequestration capacity is crucial for the sustainable use of soil in the future.

The SOC content in the soil exceeds the sum of global vegetation and atmosphere. Forest SOC pool, the world’s largest soil C pool^14^, accounts for about 73% of the global soil C pool. Its slight changes may cause significant changes in atmospheric CO_2_ concentration, directly affecting the C cycle of terrestrial ecosystems and global C balance^7,15,16^. The sequestration of SOC can help alleviate the increase in atmospheric CO_2_ content^17,18^, as most agricultural soils were in a state of organic matter consumption under traditional cultivation. It can also improve soil fertility and ensure sustainable land use^19,20^. Climate change, soil type, land management measures, vegetation types, and their interactions all affect the turnover and stability of SOC^21,22^.

Soil aggregates are usually divided into different sizes through wet sieving and dry sieving^13^, and their stability is evaluated by the distribution and different sizes of aggregates^23,24^. Changes in land use have a significant impact on the distribution and stability of soil aggregates^25,26^. Soil aggregates > 0.053 mm at the 0–20 cm depth increased after 20 years of converting farmland into forestland in southern China^27^. One study found that conversion from farmland to forestland (after 18 years) increased the soil aggregates (> 0.25 mm), MWD, and GMD but decreased soil aggregates (< 0.25 mm) at the 0–20 cm depth in the Loess Plateau, China^24^. Also some studies have indicated that after returning farmland to forests (after 20 years) decreased the MWD, GMD, and the soil aggregates (> 2 mm) at the 0–20 cm soil depth in the south China^28^. Soil aggregates were the key to all C sequestration mechanisms^29^, and SOC benefited the formation and stability of soil aggregates^30,31^. In turn, soil aggregates affect the decomposition of SOC and are an important mechanism for SOC sequestration^32^. The aggregation process of soil determines the degree of protection of SOC, and due to different protection mechanisms, the stability and retention time of SOC in macroaggregates and microaggregates were also different^33,34^. Researchers found that SOC rapidly decomposed as macroaggregates broke, suggesting that SOC’s stability was highly dependent on physical protection by macroaggregates^35^. Macroaggregate promotes the formation of internal microaggregates, which is the primary mechanism for SOC accumulation and stabilization^36^, microaggregates are important for the long-term stability of SOC^37^. However, there are differences in the results of different studies. For example, one study showed that afforestation significantly increased the soil organic carbon in aggregates of different sizes at the 0–40 cm soil depth^27^. Another study showed that afforestation only increased the soil organic carbon content in macroaggregates^38^. While considerable research has delved into the stability of SOC and its interplay with soil aggregates^24,25,39^. However, there remains a notable gap in understanding the variations in soil aggregates’ distribution, aggregate stability, and SOC following land use transitions from farmland to forest and grassland.

In the present study, we hypothesized that conversion farmland into grassland and forestland, the distribution of soil aggregates is changed, and the stability and carbon sequestration capacity of soil aggregates are improved. The objectives of this study were to (1) Determine the impact of land use pattern on soil aggregates and soil organic carbon of different particle sizes and bulk siol, (2) elucidate the relationship between SOC storage and soil aggregates’ stability. The findings of this study will underscore the importance of vegetation restoration efforts in enhancing soil quality and C sequestration potential, thus contributing to sustainable land management practices and ecological benefits.

## Materials and methods

### Study area

**Fig. 1:**
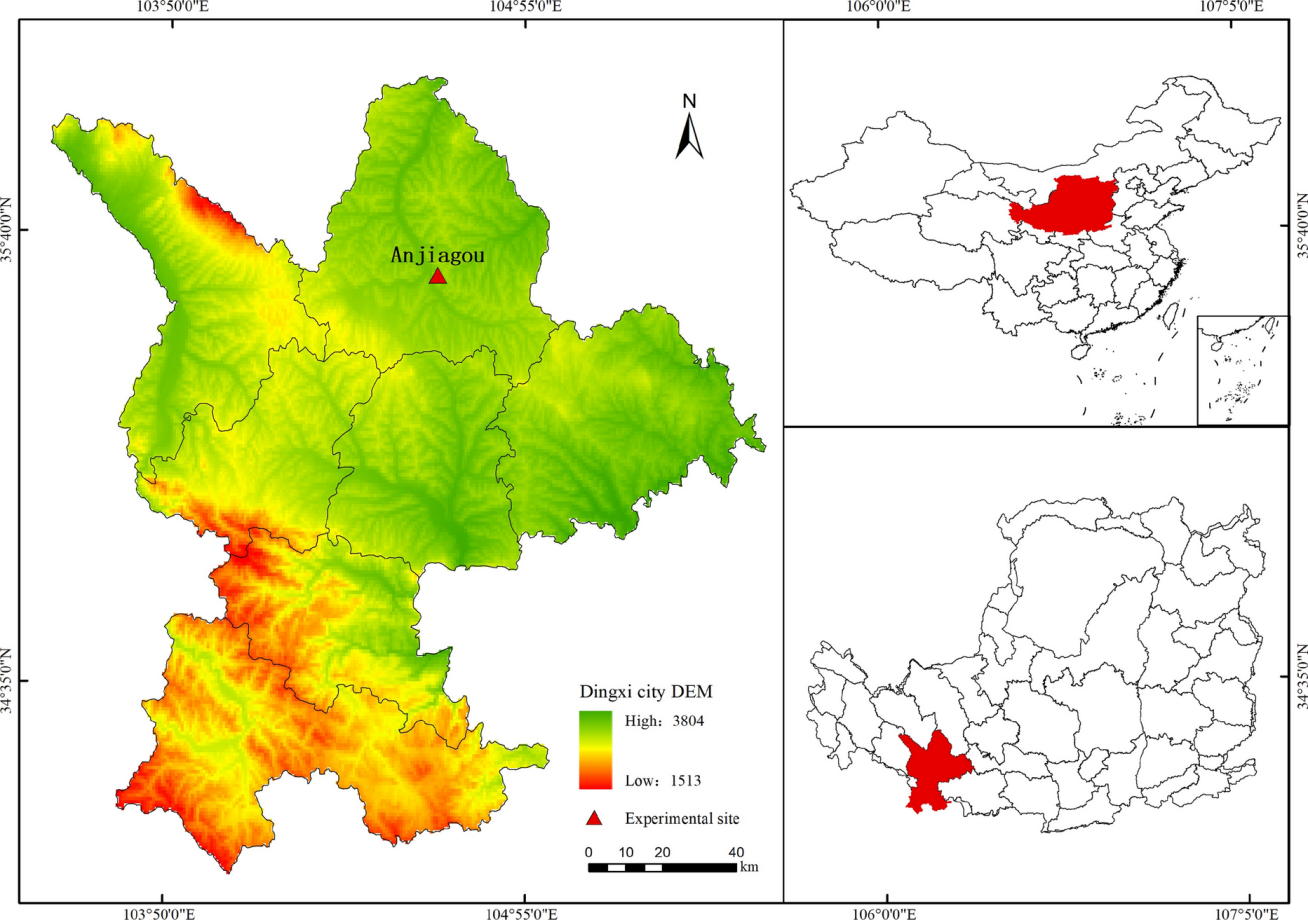
The geographical location map of the study area. Created software is ArcGIs Desktop 10.7 (version number: 10.7.0.10450, Esri Inc, All Rights Reserved, URL:http://www.esri.com).

The study was carried out in the Anjiagou watershed of Dingxi City (35°33ʹ–35°35ʹ N, 104° 38ʹ–104°40ʹ E, 1900–2250 m elevation), which was a small tributary of the Guanchuan River in the Zuli River system of the Yellow River Basin and a part of typical hilly-and-gully region of Loess Plateau, China (Fig. 1). The study area has a temperate semiarid zone climate, with a mean annual temperature of 6.3 °C, an annual sunshine duration of 2408.6 h, a frost-free period of 141 days, and an average annual precipitation (more than 60% concentrated in July to September) of 427 mm^40^. The main soil types were loess and river saline soils, categorized as Calcaric Cambisol^41^. It features weak cohesion, high infiltrability, low water retention, and is prone to erosion^24^. Before the 1990s, to meet the increasing demand for food from the rapidly growing population, the native vegetation in the region was destroyed, resulting in severe soil erosion and land degradation. In the 1990s, “the Conversion of Farmland to Forests and Grasses Project” was officially launched to protect against soil erosion, and many farmlands were abandoned and replaced by artificial forests and grasslands^42^. *Picea asperata Mast.* (PA), *Platycladus orientalis* (L.) *Franco* (PO) and *Pinus tabuliformis Carrière* are common afforestation tree species in the local area*. Medicago sativa* (MS) was a perennial herbaceous plant with strong drought resistance and adaptation to poor soils, which was widely planted in the region^43^. Moreover, the dominant species in natural grasslands (NG) include *Leontopodium leontopodioides(Willd.) Beauv., Artemisia annua L.*, *Picris hieracioides L..* The dominant species in abandoned cropland (ACL) include *Leymus secalinus (Georgi) Tzvelev., Stipa bungeana Trin., Plantago asiatica L., Setaria viridis (L.) P. Beauv.*

### Experimental design and sampling

Sampling was conducted in July 2023 (July 12th) during peak vegetative growth and species richness. Six typical land use types were selected based on a thorough investigation of land use changes in the study area: farmland (FL), *Medicago sativa* (MS), *Picea asperata* Mast. (PA), *Platycladus orientalis* (L.) Franco (PO), abandoned cropland (ACL), and natural grassland (NG). These sites were chosen to represent diverse land use scenarios while ensuring similar geographical characteristics such as elevation, slope gradient, and parent soil material. The restoration times for *Medicago sativa*, *Picea asperata* Mast., *Platycladus orientalis* (L.) Franco, and abandoned cropland were 10 years, 28 years, 10 years, and 10 years, respectively. Maize (*Zea mays* L.) and wheat (*Triticum aestivum* L.) were the main crops before the evolution from farmland to forestland or grassland, with no subsequent destruction or human disturbance noted at the sampling sites. Each land use pattern was replicated three times, with sampling sites on sloping land (< 5°) and uniform areas of 20 m × 20 m. The soil samples were collected at each land use patterns, and five points were randomly selected in an “S” shape in each sample plot, and soil samples were collected from the 0–10 cm, 10–30 cm, and 30–50 cm soil depth with a soil auger with a diameter of 5 cm, and soil samples from the same soil depth were composed of mixed soil samples. The soil aggregate samples were randomly selected from 5 points using the “five point method” in each sample plot, and the soil aggregate collected from the 0–10 cm, 10–30 cm, and 30–50 cm soil depth with an iron shovel, and soil aggregate from the same soil depth were composed of mixed soil samples. The soil samples stored in self-sealing bags and soil aggregate samples in plastic lunch boxes to prevent extrusion and shock during transportation to the laboratory. Upon arrival, roots, stones, and visible plant debris were meticulously removed by hand from the samples. The soil samples was used for the determination of soil moisture (SM) content, soil organic carbon, and pH. Soil aggregate samples was used to determine the distribution and stability of aggregates. The soil bulk density (BD) samples were randomly selected from 5 points using the “five point method” in each sample plot with a cutting ring (100 cm^3^), and soil bulk density were collected from the 0–10 cm, 10–30 cm, and 30–50 cm soil depth. After quickly measuring the fresh weight of the soil with the cutting ring on site, it is placed in a sealed bag and brought back to the laboratory for drying, weighing, and calculating the soil bulk density. The soil bulk density from the same soil depth was the average of five samples.

### Soil laboratory analysis

The undisturbed soil in plastic lunch boxes was brought back to the lab and was gently divided into small pieces (near 1 cm), and roots, stones and other debris were removed and naturally kept dry. Then, we weighed a 500 g sample and used a dry sieve method to obtain the aggregates of < 0.25 mm, 0.25–0.5 mm, 0.5–1 mm, 1–2 mm, 2–5 mm, and > 5 mm. According to the proportion of each aggregate size, 50 g of a mixed soil sample, including different aggregate fractions, were matched, and a wet sieve method was used to obtain water-stable aggregates of different sizes^44,45^. The processed samples underwent oven drying at 50 °C for 48 h until reaching constant weight to determine the content of each aggregates. Soil aggregate and air-dried soil samples were ground to pass through a 0.25 mm sieve and stored in plastic bags for SOC and pH analysis. SOC content was measured using the K_2_Cr_2_O_7_–H_2_SO_4_ oxidation method described^39^. Soil pH was determined in a 1:2.5 soil/water solution using a PHS-3C instrument^39^. The soil moisture content was determined by drying method. Soil BD was determined by the cutting ring method^46^.

### Soil mean weight diameter (MWD) and geometric mean diameter (GMD)

The mean weight diameter (MWD) and geometric mean diameter (GMD) were used to evaluate soil aggregate stability under different land use patterns. The two parameters were calculated using the following two formulas^24,39^.

$$\begin{aligned} {\text{MWD}} & = \sum\limits_{i = 1}^{{\text{n}}} {xiwi} \\ {\text{GMD}} & = {\text{exp}}\left[ {\frac{{\sum\nolimits_{i = 1}^{{\text{n}}} {(wi\ln xi)} }}{{\sum\nolimits_{i = 1}^{n} {wi} }}} \right] \\ \end{aligned}$$

where *x*_*i*_ was the mean diameter (mm) of different soil aggregate-size, *w*_*i*_ was the content (%) of soil aggregates in the *i*th size class to the total mass of the samples, and *n* was the number of different size soil aggregates, and ln*x*_*i*_ was the base-e natural logarithm of the mean diameter for different aggregate-size class.

The contribution of soil different aggregates with particle sizes to the total carbon contents of soil after changes in land use patterns as following equations^39^.

$$\begin{aligned} \Delta \mathop C\nolimits_{i} & = \left( {\mathop C\nolimits_{{D{\text{i}}}} \times \mathop w\nolimits_{Di} } \right) - \left( {\mathop C\nolimits_{Fi} \times \mathop w\nolimits_{Fi} } \right) \\ \mathop G\nolimits_{i} & = \left( {\frac{{\Delta \mathop C\nolimits_{i} }}{{\mathop C\nolimits_{D} - \mathop C\nolimits_{F} }}} \right) \times 100 \\ \end{aligned}$$

where *ΔC*_*i*_ was the difference in SOC content in soil aggregate *i* between the resumed forests or grasslands from farmlands and the FL;* C*_*Di*_ was the SOC content in soil aggregate *i* under resumed forests or grasslands from farmlands, and *w*_*Di*_ was the content (%) of soil aggregate *i* in bulk soil under resumed forests or grasslands from farmlands. *C*_*Fi*_ was the SOC content in soil aggregate *i* under FL, and *w*_*Fi*_ was the content (%) of soil aggregate i in bulk soil under FL. *G*_*i*_ was the contribution (%) of soil aggregate *i* to SOC contents in bulk soil when FL was converted to the forests or grasslands, *C*_*D*_ was the SOC content in bulk soil under forests or grasslands, and *C*_*F*_ was the SOC content in bulk soil under FL.

### Statistical analysis

We used Microsoft Excel 2019 computer software to organize the data, and all data were analyzed using SPSS 19.0 statistical software (SPSS Inc., Chicago, IL, USA). Before analysis, all data underwent assessments for normality and homogeneity of variance. The distribution of soil aggregates of different sizes, MWD, GMD, pH, SM, BD, and SOC across various land uses was compared through a one-way variance analysis (ANOVA). In cases where ANOVA indicated a significant effect, post hoc least significant difference (LSD, P < 0.05) tests were employed for pairwise comparisons. Use the plugin of OriginPro 2021 software (Correlation Plot, version v1.10) to correlation matrices between soil aggregate stability indices (MWD and GMD) and various soil physical–chemical properties were generated based on Pearson correlation coefficients, with significance levels set at P < 0.05 and P < 0.01. We used the plugin of OriginPro 2021 software (Paired Comparison Plotv 3.60) plot comparison chart of MWD and GMD pairing, and use he plugin of OriginPro 2021 software (multi factor group bar chart) plot comparison chart of SOC in different particle size aggregate and bulk soil pairing. We also used OriginPro 2021 software for regression analysis on soil organic carbon and organic carbon in aggregates of different particle sizes.

## Results

### Distribution characteristics of soil aggregates

Land use patterns had significantly affect the distribution of soil aggregates (Table 1). Microaggregates (< 0.25 mm) dominate in aggregate size of different soil depths of FL. However, microaggregates were significantly decreased by 16.66–70.82%, 39.12–83.17%, 0.48–18.20%, 45.74–72.22%, and 7.60–41.49% after FL transforms into grassland (MS, NG, and ACL) and forestland (PA, PO), respectively. With the extension of transition time, soil macroaggregates (> 2 mm) significantly increased and soil microaggregates significantly decreased (*P* < 0.05), the distribution of soil aggregates no obvious rules between soil depths. During the same period of restoration, the proportion of aggregates (> 2.0 mm) in 0–10 cm and 10–30 cm soil depths was MS > PO > ACL, and the proportion of aggregates (< 0.25 mm) were MS < PO < ACL, and there was no obvious rules of aggregates (0.25–2.0 mm) (*P* < 0.05). At three soil depths, the proportion of macroaggregates in forestland and grassland (PA, NG, MS, PO, and ACL) was significantly higher than those in FL, while the proportion of microaggregates were significantly lower than those in FL. Among them, the macroaggregates in PA and NG treatments were significantly higher than those in other treatments, and the proportion of microaggregates was significantly lower than those in other treatments (*P* < 0.05).

**Table 1:** Distribution of soil aggregates under different land uses (means ± SDs).

Land uses	Aggregate-size distribution (g)					
	> 5 mm	2–5 mm	1–2 mm	0.5–1.0 mm	0.25–0.5 mm	< 0.25 mm
Soil depth 0–10 cm						
FL	0.48 ± 0.05Cd	0.89 ± 0.05Cc	1.72 ± 0.09Bc	1.79 ± 0.22Bb	2.38 ± 0.23Aab	42.73 ± 0.33Aa
MS	26.29 ± 1.50Ab	3.84 ± 0.29Aa	2.32 ± 0.12Bb	2.64 ± 0.17Aa	2.44 ± 0.20Aab	12.47 ± 0.94Cd
PA	32.06 ± 1.57Aa	2.22 ± 0.18Bb	1.72 ± 0.16Bc	1.10 ± 0.10Bc	1.04 ± 0.16Cd	11.87 ± 1.45Cd
PO	18.06 ± 2.75Bc	1.85 ± 0.14Bb	2.19 ± 0.11Bb	1.37 ± 0.10Bbc	1.54 ± 0.11BCcd	25.00 ± 2.76Bc
ACL	4.98 ± 0.13Cd	1.82 ± 0.17Bb	1.96 ± 0.2Bbc	1.76 ± 0.06Bb	2.86 ± 0.10Aa	36.61 ± 0.21Ab
NG	29.82 ± 0.47Aab	3.52 ± 0.15Aa	4.71 ± 0.31Aa	2.66 ± 0.31Aa	2.10 ± 0.18ABbc	7.19 ± 0.88Ce
Soil depth 10–30 cm						
FL	0.78 ± 0.04De	1.29 ± 0.10Cc	2.26 ± 0.25BCbc	4.00 ± 0.19Aa	6.02 ± 0.26Aa	35.65 ± 0.38Aa
MS	11.57 ± 1.18BCbc	2.36 ± 0.22Bb	2.23 ± 0.17BCc	2.69 ± 0.22Cc	4.49 ± 0.25Bb	26.66 ± 1.96Bb
PA	27.21 ± 2.48Aa	1.64 ± 0.08Cc	1.00 ± 0.08Dd	1.46 ± 0.22Dd	1.35 ± 0.22De	17.34 ± 2.02Cc
PO	8.98 ± 0.47BCcd	1.29 ± 0.14Cc	1.67 ± 0.22CDc	2.62 ± 0.06Cc	2.50 ± 0.26Cd	32.94 ± 1.09Aa
ACL	6.93 ± 0.32Cd	1.49 ± 0.20Cc	2.88 ± 0.21Bb	1.29 ± 0.15Dd	1.58 ± 0.10De	35.82 ± 0.33Aa
NG	13.66 ± 1.56Bb	4.53 ± 0.14Aa	4.51 ± 0.31Aa	3.47 ± 0.17Bb	3.65 ± 0.14BCc	20.18 ± 1.58Cc
Soil depth 30–50 cm						
FL	0.09 ± 0.01Cd	0.95 ± 0.06Bb	0.70 ± 0.03Cd	1.16 ± 0.11Dd	2.27 ± 0.17Bbc	44.84 ± 0.30Aa
MS	6.11 ± 1.02Bc	1.50 ± 0.19Bb	1.02 ± 0.06BCc	1.49 ± 0.18CDcd	2.51 ± 0.13Bb	37.37 ± 1.29Bb
PA	19.87 ± 1.18Aa	1.22 ± 0.10Bb	0.91 ± 0.07BCcd	1.85 ± 0.20Cc	1.82 ± 0.14Bc	24.33 ± 1.36De
PO	6.56 ± 0.37Bbc	1.06 ± 0.10Bb	1.36 ± 0.14ABb	2.87 ± 0.09Bb	4.71 ± 0.25Aa	33.44 ± 0.77Cc
ACL	7.71 ± 0.32Bbc	1.03 ± 0.05Bb	1.12 ± 0.08BCbc	1.56 ± 0.12CDcd	1.90 ± 0.21Bc	36.68 ± 0.30BCb
NG	8.16 ± 0.80Bb	6.24 ± 0.59Aa	1.76 ± 0.18Aa	4.00 ± 0.09Aa	2.55 ± 0.14Bb	27.30 ± 1.36Dd

Different uppercase letters and lowercase letters indicate significant (*P* < 0.05) differences among different land use patterns. < 0.25 mm, 0.25–0.5 mm, 0.5–1.0 mm, 1–2 mm, 2–5 mm and > 5 mm represent the particle size distribution of aggregates. FL, MS, PA, PO, ACL and NG, represent Farmland, *Medicago sativa*, *Picea asperata* Mast., *Platycladus orientalis* (L.) Franco, Abandoned cropland and Natural grassland, respectively.

### Stability of soil aggregates

**Fig. 2:**
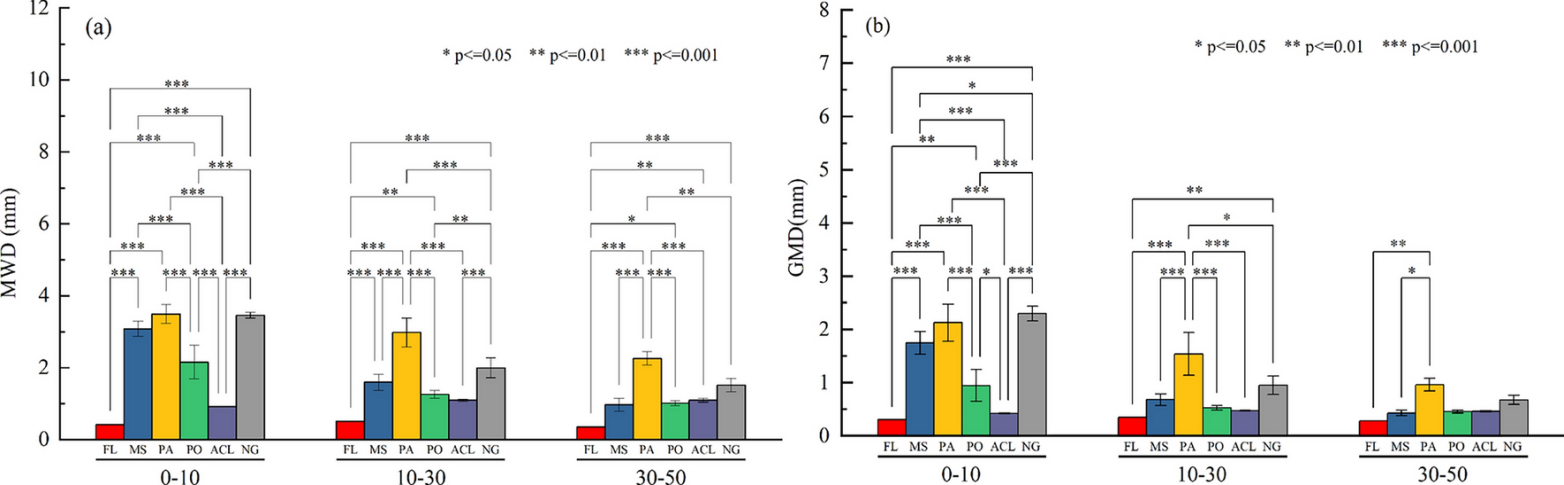
The average weight diameter (**a**) and geometric average diameter (**b**) of soil aggregates at different depths under different land use patterns. Values are given as means ± standard error (n = 3). Different * indicate significant differences (* *P* ≤ 0.05, ** *P* ≤ 0.01, *** *P* ≤ 0.001) among different land use patterns. FL, MS, PA, PO, ACL and NG, represent Farmland, *Medicago sativa*, *Picea asperata* Mast., *Platycladus orientalis* (L.) Franco, Abandoned cropland and Natural grassland, respectively.

The mean weight diameter (MWD) and geometric mean diameter (GMD) of soil aggregates considerably varied among the six land uses (Fig. 2). Similar changing trends for the MWD and GMD were observed among the three soil depths, and the highest values were observed under the PA treatment (PA > NG > MS > PO > ACL > FL). At the three depths, the average values of MWD and GMD under FL were significantly lower than those under MS, PA, PO, and NG (*P* < 0.05). Among the 5 vegetation restoration measures, the MWD values were significantly higher than PO and ACL under the PA and NG at the three different soil depths. The GMD values were significantly higher than PO and ACL under the NG at the 0–10 cm soil depths (*P* < 0.01). However, the GMD values were significantly higher than MS, PO, ACL, and NG under the PA at 10–30 cm and 30–50 cm (*P* < 0.05). The variation range of MWD were 0.42 mm to 3.50 mm, 0.52 mm to 2.98 mm, and 0.35 mm to 2.26 mm at the three depths, respectively, and the value range of GMD from 0.30 mm to 2.30 mm, 0.35 mm to 1.54 mm, and 0.28 mm to 0.96 mm at the three depths, respectively, which indicated that the soil water-stable aggregates in topsoil were more stable than those of the subsoil.

### SOC content in bulk soil and in different soil aggregates

**Fig. 3:**
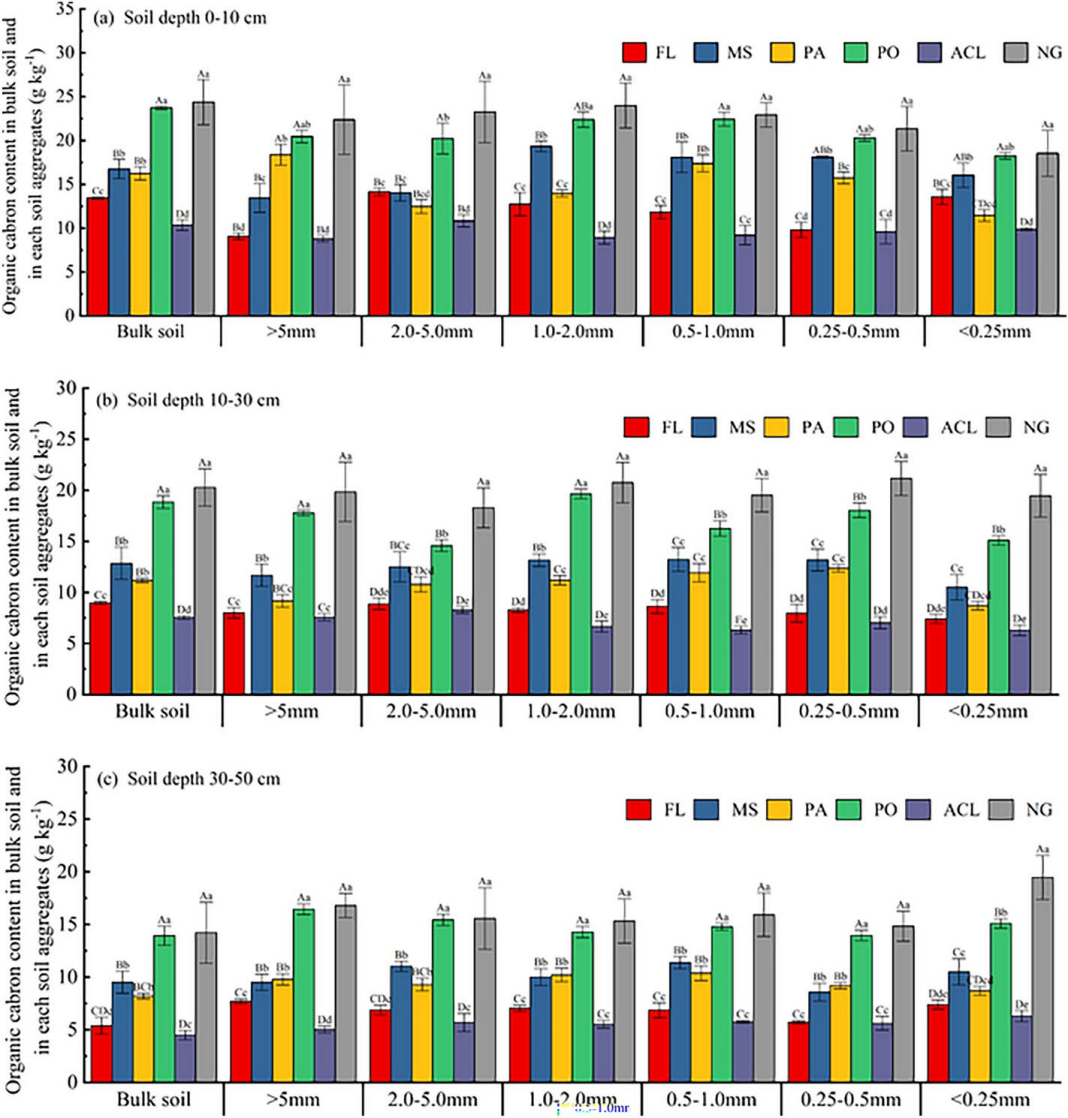
Soil organic carbon (SOC) content in soil and aggregates at different depths under different land use patterns. Values are given as mean ± standard error (n = 3). Different uppercase letters and lowercase letters indicate significant (*P* < 0.05) differences among different land use patterns. FL, MS, PA, PO, ACL and NG, represent Farmland, *Medicago sativa*, *Picea asperata* Mast., *Platycladus orientalis* (L.) Franco, Abandoned cropland and Natural grassland, respectively.

Among the three soil depths, the SOC content in bulk soil and soil aggregates was the highest under NG and lowest under ACL. The SOC content in bulk soil and soil aggregates was significantly higher than FL, MS, PA, and ACL under NG, and significantly higher than FL and ACL under PO and NG (*P* < 0.05). At the 0–10 cm soil depth. In addition, The SOC content were significantly higher than FL and ACL under MS and PA in soil aggregates (> 5 mm, 0.5–1.0 mm, 0.25–0.5 mm), and significantly higher than FL and ACL under MS in soil aggregates (> 2 mm and < 0.25 mm) (*P* < 0.05) (Fig. 3a). At the soil depths of 10–30 cm and 30–50 cm, the SOC content in bulk soil and soil aggregates were significantly higher than FL, MS, PA, and ACL under NG, and significantly higher than FL and ACL under MS (*P* < 0.05). However, the SOC content in soil aggregates (> 5 mm and 1–2 mm) treatment were significantly higher than PA under MS at 10–30 cm soil depth (Fig. 3b, c). In soil depths 0–10 cm, 10–30 cm, and 30–50 cm, the average SOC content in the bulk soil and soil aggregates of > 5 mm, 2–5 mm, 1–2 mm, 0.5–1 mm, 0.25–0.5 mm and < 0.25 mm was 17.49, 15.43, 15.84, 16.90, 16.99, 15.82 and 14.63 g kg^−1^, 13.28, 12.34, 12.22, 13.28, 12.64, 13.29 and 11.24 g kg^−1^, and 10.98, 9.29, 10.88, 10.64, 10.39, 10.84 and 9.64 g kg^−1^, respectively. Compared with the SOC content under FL in three different soil depths, the average SOC content in the bulk soil and soil aggregates of > 5 mm, 2–5 mm, 1–2 mm, 0.5–1 mm, 0.25–0.5 mm and < 0.25 mm under the forestland and grassland increased by 4.83, 7.62, 2.02, 4.98, 6.16, 7.21 and 1.25 g kg^−1^, 5.16, 5.21, 4.01, 6.01, 4.83, 6.39 and 4.62 g kg^−1^ and 3.62, 4.68, 3.81, 4.53, 4.04, 4.78 and 4.71 g kg^−1^, respectively.

### Contributions of different soil aggregates to SOC sequestration in bulk soil after afforestation

Compared with FL, the SOC content in bulk soil under MS, PA, PO, and NG increased by 24.57%, 20.73%, 76.25%, and 81.05%, respectively, at the 0–10 cm depth, by 43.10%, 24.39%, 109.91%, and 125.91%, at the 10–30 cm depth, and by 33.92%, 26.14%, 102.93% and 90.59%, respectively, at the 30–50 cm depth. However, ACL treatment decreased by 23.06%, 16.04%, and 26.73% in 0–10 cm, 10–30 cm, and 30–50 cm soil depths, respectively (Table 2). Significant differences were in the contribution rates of different particle size aggregates to soil SOC content after changes in land use patterns (Table 2). When FL was transformed into MS, PA, PO, and NG, the soil aggregates (> 5 mm and 2–5 mm) in the three soil depths all had a positive contribution to the increase of soil SOC content, while the soil aggregate (< 0.25 mm) had the opposite effect in 0–10 cm depth. However, when FL was transformed into ACL, the contributions of the soil aggregates (> 5 mm and 2–5 mm) in 0–10 cm soil depth, and soil aggregates (2–5 mm) in 10–30 cm to SOC content were negative, but the soil aggregate (< 0.25 mm) had the positive effect (Table 2). After FL was transformed into forestland and grasslands, the contributions of soil aggregates (> 5 mm) to soil SOC were 211.29%, 419.49%, 71.16%, − 25.39%, and 121.51%, respectively, and the contributions of soil aggregates (< 0.25 mm) to soil SOC were − 229.97%, − 318.56%, − 24.20%, 141.20%, and − 81.97% in the 0–10 cm soil depth, respectively. The contributions of soil aggregates (> 5 mm) to soil SOC content were 66.60%, 125.56%, 79.21%, 23.89%, and 136.86%, respectively, and the contributions of soil aggregates (< 0.25 mm) were 8.62%, − 102.77%, 47.39%, 52.79%, and 22.91% in the 10–30 cm soil depth, respectively. However, the contributions of soil aggregates (> 5 mm) were 42.66%, 120.15%, 67.36%, 25.28%, and 85.41, respectively, while the contributions of soil aggregates (< 0.25 mm) were 46.92%, − 31.89%, 51.22%, 47.62%, and 41.08%, in the 30–50 cm soil depth, respectively (Table 2).

**Table 2:** Influence of returned farmland to forestland or grassland on SOC content in bulk soil and the contributions of different soil aggregates to SOC sequestration in bulk soil.

Land use change from FL to-	SOC changes in bulk soil	G_i_ (%)					
	(g kg^−1^)	> 5 mm	2–5 mm	1–2 mm	0.5–1 mm	0.25–0.5 mm	< 0.25 mm
Soil depth 0–10 cm							
MS-FL	3.31	211.29	25.03	13.92	16.05	12.59	− 229.97
PA-FL	2.79	419.49	10.83	1.48	− 1.53	− 5.07	− 318.56
PO-FL	10.26	71.16	4.82	5.28	1.84	1.52	− 24.20
ACL-FL	− 3.10	− 25.39	− 4.62	2.84	3.20	− 2.65	141.20
NG-FL	10.91	121.51	12.70	16.71	7.29	3.95	− 81.97
Soil depth 10–30 cm							
MS-FL	3.87	66.60	9.37	5.51	0.53	5.80	8.62
PA-FL	2.19	125.56	5.75	− 6.82	− 15.66	− 28.51	− 102.77
PO-FL	9.87	79.21	1.50	2.86	1.62	− 0.56	47.39
ACL-FL	− 1.44	23.89	− 1.26	− 0.67	36.59	51.12	52.79
NG-FL	11.31	136.86	12.62	13.24	5.87	5.20	22.91
soil depth 30–50 cm							
MS-FL	2.70	42.66	5.14	4.76	5.04	9.56	46.92
PA-FL	2.08	120.15	4.47	3.53	10.32	3.17	− 31.89
PO-FL	8.20	67.36	2.46	3.96	7.99	13.16	51.22
ACL-FL	− 2.13	25.28	2.01	− 1.47	− 0.47	4.38	47.62
NG-FL	7.22	85.41	27.02	6.24	14.71	6.92	41.08

G_i_: contributions of different soil aggregates to SOC sequestration in bulk soil after afforestation. < 0.25 mm, 0.25–0.5 mm, 0.5–1.0 mm, 1–2 mm, 2–5 mm and > 5 mm represent the particle size distribution of aggregates. FL, MS, PA, PO, ACL and NG, represent Farmland, *Medicago sativa*, *Picea asperata* Mast., *Platycladus orientalis* (L.) Franco, Abandoned cropland and Natural grassland, respectively.

### The correlations between the soil aggregate stability and soil hysicochemical properties

**Fig. 4:**
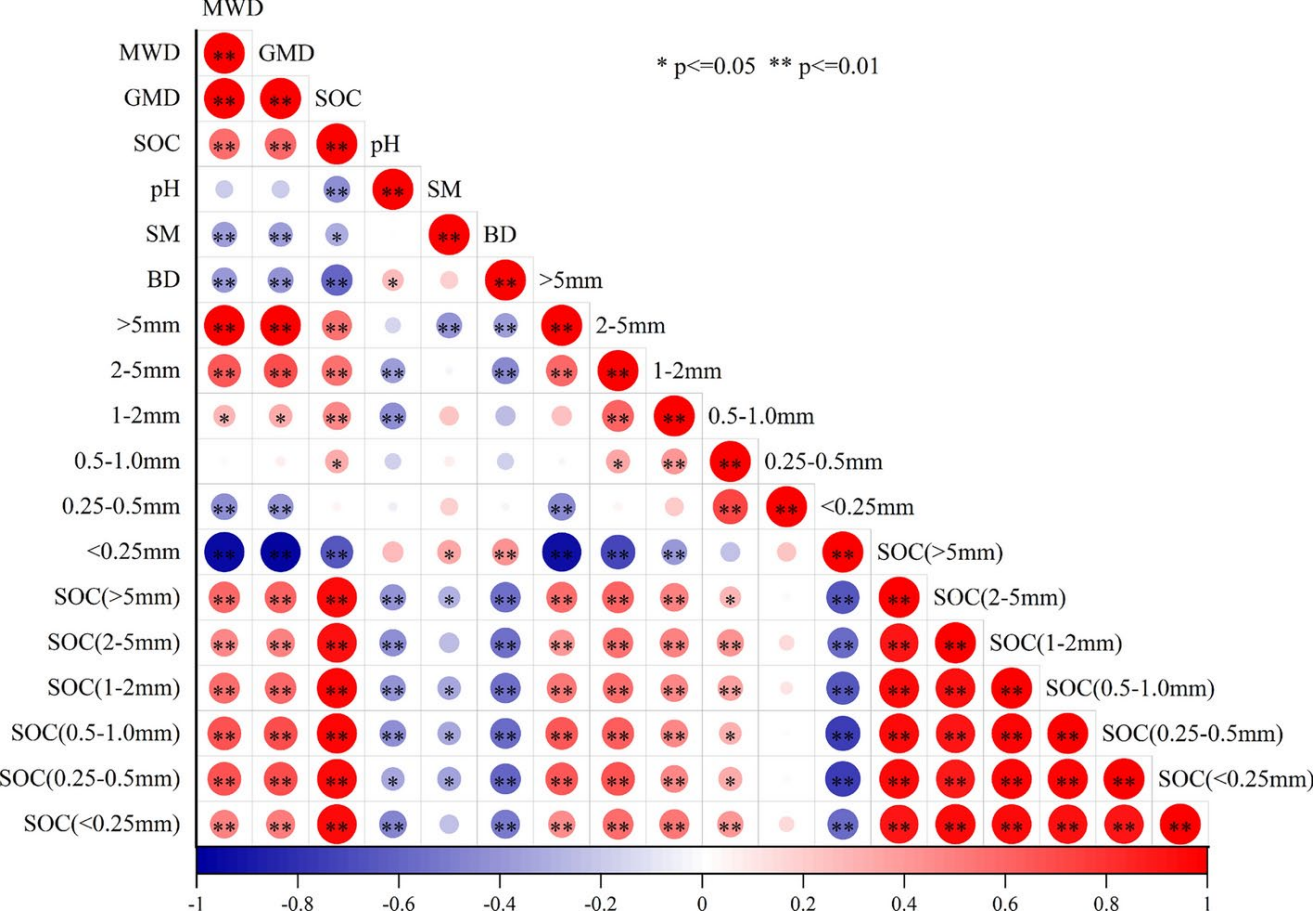
Correlation analysis between soil organic carbon and soil properties in bulk soil and aggregates with different particle sizes. * and ** indicate *p* ≤ 0.05, and *p* ≤ 0.01. MWD and GMD represent mean weight diameter and geometric mean diameter. < 0.25 mm, 0.25–0.5 mm, 0.5–1.0 mm, 1–2 mm, 2–5 mm and > 5 mm represent the particle size distribution of aggregates. SOC, SOC(< 0.25 mm), SOC(0.25–0.5 mm), SOC(0.5–1.0 mm), SOC(1–2 mm), SOC(2–5 mm) and SOC(> 5 mm) represent the SOC content in bulk soil and aggregates of different particle sizes.

**Fig. 5:**
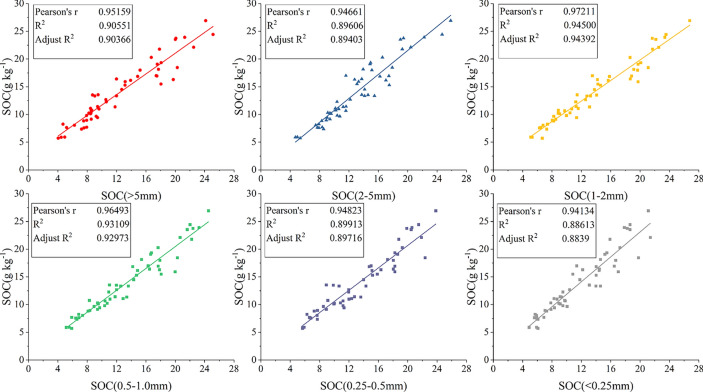
Relationship of soil organic carbon (SOC) and soil organic carbon (SOC) in aggregates of different particle sizes. SOC(< 0.25 mm), SOC(0.25–0.5 mm), SOC(0.5–1.0 mm), SOC(1–2 mm), SOC(2–5 mm) and SOC(> 5 mm) represent the SOC content in bulk soil and aggregates of different particle sizes.

The MWD was positively correlated with the GMD (Fig. 4). The MWD and GMD were positively correlated with the soil aggregates of > 1 mm, and SOC in bulk soil and aggregates soil, but negatively correlated with the soil aggregates of < 0.5 mm, soil pH, soil moisture and soil bulk density (Fig. 4). The SOC content in bulk soil was positively correlated with SOC in soil aggregates (< 0.25 mm (R^2^ = 0.88613), 0.25–0.5 mm (R^2^ = 0.89913), 0.5–1.0 mm (R^2^ = 0.93109), 1–2 mm (R^2^ = 0.94500), 2–5 mm (R^2^ = 0.89606), > 5 mm (R^2^ = 0.90551)) and soil aggregates (> 0.5 mm) but negatively correlated with soil aggregates (< 0.25 mm), soil pH, soil moisture and soil bulk density (Figs. 4 and 5). SOC content in aggregates was positively correlated with soil aggregates (> 0.5 mm), but negatively correlated with soil pH, soil moisture and soil bulk density. (Fig. 4). Soil aggregates (> 0.5 mm) was negatively correlated with soil pH and soil bulk density, soil aggregates (> 5 mm) was negatively correlated with soil moisture. However, soil aggregates (< 0.25 mm) was positively correlated with soil pH, soil moisture and soil bulk density (Fig. 4).

## Discussion

The results of this study align with the initial hypothesis regarding the significant variations in soil aggregates’ distribution, aggregate stability, and carbon sequestration capacity of soil aggregates following land use transitions from farmland (FL) to forest and grassland in the Loess Plateau, China. Moreover, the current research findings support the expectation that farmland transitioning to forest and grassland would enhance SOC storage and promote the stability of soil aggregates. This was attributed to the increased vegetation cover and restoration efforts associated with such land use changes. The observed changes in soil aggregates’ distribution and stability, coupled with the noticeable increase in SOC content, underscore the positive impact of vegetation restoration on soil quality and carbon sequestration potential. These outcomes validate the importance of sustainable land management practices, particularly in ecologically sensitive regions like the Loess Plateau, where soil erosion and degradation are prevalent concerns. These findings have significant implications for policymakers, land managers, and conservationists striving to balance agricultural productivity with environmental conservation goals.

### Effects of vegetation restoration measures on the distribution of aggregate fractions

Significant increases in aggregates of > 5 mm content and significant decreases in aggregate of < 0.25 mm content were observed and no obvious pattern in the changes of 0.25–5 mm aggregates under the returning farmland to forests and grasslands in the present study (Table 1). These observations suggest that afforestation and grass planting initiatives in former farmlands have facilitated the formation of macroaggregates, thereby enhancing soil structure in the Loess Plateau, China. This similar finding was similar to the results of Yu et al.^39^ study conducted in southwest China, and also supported the relevant study of Dou et al*.*^44^ conducted in the central part of the Loess Plateau. They indicate that the proportion of macroaggregates in farmland soil was significantly lower than that in forestland and grassland, the dominant factors were tillage operations and precipitation shocks^24,44,47^. Ongoing tillage activities may disrupt the macroaggregate structure in the soil^48^ or impede the natural transformation process of microaggregates into macroaggregates^23,49^. Furthermore, prolonged farming activities result in the constant mixing of soil layers, particularly under rain erosion, which proportion of macroaggregates more likely to be destroyed^50^, thereby relatively increased the proportion of small aggregates in the soil^50,51^. Upon transitioning farmland back to forests and grasslands, there was a notable increase in aggregates larger than 5 mm and a decrease in aggregates smaller than 0.25 mm. This phenomenon can be attributed to several factors related to vegetation type, root systems, and litter. On the one hand, it is because the vegetation restoration process increases vegetation coverage and litter content, reduces soil water evaporation, and increases humus content^52^, On the other hand, the increase in root biomass leads to the concretion and release of secretions by the roots, enhancing the soil’s resistance to erosion and the ability of microaggregates to cement , thereby increasing the proportion of macroaggregate in the soil. At the same time, it is also related to the content of soil organic carbon, which is conducive to the formation and stability of soil aggregates^30,31^. Conversely, soil aggregates influence SOC decomposition and serve as an essential mechanism for SOC sequestration^32^. This is consistent with the significant positive correlation between soil organic carbon and soil aggregates > 0.5 mm, and the significant negative correlation with soil aggregates < 0.25 mm in this study (Fig. 4).

### Response of aggregate stability to different vegetation restoration measures

The MWD and GMD of soil aggregates are pivotal indicators for assessing their stability and aggregation capacity. Higher values of these indices signify stronger aggregate stability and enhanced aggregation capabilities^44,53^. Consequently, aggregate stability variations reflect their composition differences^54^. The findings of this study demonstrate that, compared to farmland (FL), the MWD and GMD of soil aggregates significantly improved (*P* < 0.05) with various vegetation restoration measures, indicating enhanced soil structure and stability through vegetation restoration. Specifically, the soil aggregate stability of forestland and natural grassland restored for 28 years showed the highest improvement, while abandoned cropland exhibited the lowest stability. This was due to the higher plant species diversity, root biomass associated with natural restoration measures, and increased years of artificial vegetation restoration, resulting in a greater amount of easily decomposable plant litter and root exudates^55^. Moreover, plant litter is a crucial source of soil organic matter, providing a conducive environment for soil microorganisms post-decomposition. Microbial activity produces substances that were conducive to soil cementation could be produced, and the combined effect with root exudates promoted the aggregation of small soil particles into larger aggregates^24^, thereby effectively enhancing the stability of soil aggregate. Notably, the study observed the strongest stability of the soil surface layer under different vegetation restoration measures, possibly due to higher moisture levels, litter content, and temperature, etc. in the soil surface layer, facilitating increased soil organic matter content and promoting the reproduction and metabolism of soil microorganisms^56^. The significant positive correlation between soil aggregate stability and soil organic carbon, and significant negative correlation (*P* < 0.05) with soil moisture and soil bulk density in this study (Fig. 4) corroborates these findings. Additionally, the relatively low stability of soil aggregates in abandoned land aligns with prior research results^44^, emphasizing the critical role of vegetation restoration measures in enhancing soil structure and stability.

### Effects of vegetation restoration measures on the soil organic carbon content

SOC is pivotal in regulating the global carbon balance, influenced by various factors such as land use, climate change, and cultivation methods^15,21^. In the current study, SOC content in grassland and forest land was significantly higher than that under farmland (FL) and abandoned cropland (ACL) (Fig. 3). The SOC content under different vegetation restoration measures followed this order: NG > PO > MS > PA > FL > ACL. Continuous cultivation of farmland soil leads to the destruction of soil aggregate structure, which in turn affects the accumulation of SOC^57^. However, as farmland transformed into grasslands and forests, increased vegetation coverage, plant species, litter, root biomass, and root exudates in the soil can not only increase the input of SOC, but also reduce the loss of SOC^25^, resulting in higher SOC content. This aligns with research findings in the Loess Plateau^25^ and southwest China region^39^. Different agroforestry systems can increase SOC storage globally, such as in the Indo-Gangetic Plains in Bihar, India^58^. Kim et al.^20^ demonstrated in a global review that after farmland was restored to secondary forests, soil SOC increased by 193%. The potential for soil carbon sequestration was influenced by many factors, including the soil’s background carbon content, which significantly impacts later carbon sequestration capacity. The average SOC content in FL soil at a depth of 0–50 cm in this study region was 9.47 g kg^−1^, significantly lower than in karst regions of Southwest China^39,59^ and the Loess Plateau hilly regions of North Shaanxi^44^, compared with FL, the SOC content in soil under NG, MS, PO, and PA was significantly increased (*P* < 0.01), indicating enormous SOC sequestration potential in the study region post-farmland conversion. However, the average SOC content in the 0–50 cm soil of ACL was 7.42 g kg^−1^, slightly lower than FL. That may be due to the lack of exogenous nutrient input in the soil post-abandonment, hindering SOC accumulation. Additionally, the predominance of natural weeds in ACL soil, limited total litter and root biomass in the short term, and seasonal growth leading to soil nutrient consumption contribute to ACL’s lower SOC content than FL.

The findings of this study revealed a significant positive correlation between soil SOC and aggregates (> 0.5 mm), as well as SOC in aggregates, while a negative correlation was observed with aggregates (< 0.25 mm), soil pH, soil moisture and soil bulk density(Fig. 4). Vegetation restoration resulted in a significant increase in aggregates (> 5 mm) and a corresponding decrease in aggregates (< 0.25 mm) (Table 1), indicating the positive role of macroaggregates in SOC accumulation (Table 2). This underscores the potential of vegetation restoration to facilitate the conversion of soil microaggregates into larger macroaggregates, with aggregates (> 0.5 mm) playing a crucial role in soil organic carbon sequestration^39,44^. Researchers suggest that organic carbon sequestration is closely linked to aggregate size^15^, with particulate SOC and mineral-associated soil organic carbon in macroaggregates serving as significant sources of SOC^60^. Our research also confirms this viewpoint (Fig. 5). Studies also indicate that the influx of organic matter from vegetation into the soil primarily drives the increase in soil macroaggregate SOC^25,61^. Furthermore, during vegetation restoration, the combined effects of plant roots and root exudation transform microaggregates into macroaggregates through cementation, thereby safeguarding SOC^62^.

In this study, the surface soil (0–10 cm) exhibited higher SOC content compared to the subsoil (10–50 cm), consistent with the consensus among researchers^24,57^. Among the different vegetation restoration measures, soils and aggregates under NG, PO, and MS demonstrated significantly higher SOC content than FL and ACL (Fig. 3). This disparity can be attributed to the substantial accumulation of fresh organic matter in the surface soil during vegetation restoration, primarily derived from plant biomass, litter, and roots^25^, and enhanced fine root system, with grasslands exhibiting higher fine root biomass than forestlands during the same restoration period. This increased fine root biomass contributes to carbon reserves through root decomposition and the release of root exudates^33,63^.

Overall, although returning FL to forestland and grassland significantly increased the SOC content in bulk soil and different soil aggregates, the SOC in soil and soil aggregates under NG and PA were significantly higher than those under MS and PO (Fig. 3). These results indicated that conversion from FL to NG and PA was more beneficial for soil carbon sequestration than the MS and PO in Loess Plateau, China. However, the soil macroaggregate content, MWD, and GMD were higher under PO and NG than under PA and MS, showing that the soil structure was more reasonable and the ability to resist soil erosion was the strongest under PO and NG. Therefore, to improve soil and water conservation capacity and increase soil carbon sequestration in the Loess Plateau, converting FL to PO and NG may be the best choice for improving the ecological environment. This study also determined soil aggregate stability and SOC at 0–50 cm depths. Future research should investigate changes in soil aggregate stability and SOC at deeper soil depths due to afforestation.

## Conclusions

The results of this study clearly showed that the distribution of soil aggregates, the stability of aggregates and the content of organic carbon in aggregates were significantly affected by the conversion of farmland to grassland and forestland. Afforestation increased the aggregate (> 5 mm) content and decreased the aggregate (< 0.25 mm) content. The higher values of GMD and MWD were found under PA, NG, MS, PO and ACL than under FL, indicating that returned farmland to forestland and grassland enhanced the soil aggregates stability. The positive correlations among aggregate stability indices, soil aggregates (> 1 mm) and SOC content in soil aggregates and bulk soil indicated that macroaggregate content and SOC content were the main driving factors of soil aggregate stability. Although the four land use patterns of PA, NG, MS, and PO increased the SOC content in aggregates and bulk soil, but the SOC accumulated by aggregates (> 5 mm) higher than that of other particle size aggregates. > 5 mm (104.74%), 2–5 mm (7.86%), 1–2 mm (4.76%), 0.5–1 mm (6.23%), and 0.25–0.5 mm (5.37%) aggregates were the main contributors to the increase SOC content in bulk soil after returning farmland to forestlands and grasslands, while < 0.25 mm (− 21.97%) aggregates had a negative effect after returning farmland to forestlands and grasslands. While the SOC content in different particle size aggregates under NG and PA is significantly higher than that under MS and PO, but the macroaggregate content, MWD, and GMD under PO and NG was higher than those under PA and MS, indicating that PO and NG were more conducive to improving soil carbon sequestration capacity and soil and water conservation capacity. We believe that this study can provide a theoretical basis for adjusting land use patterns and promoting sustainable land use.

## Data Availability

The datasets used during the current study available from the corresponding author on reasonable request.
