# Author Correction: Response of soil organic carbon and soil aggregate stability to changes in land use patterns on the Loess Plateau

**DOI:** 10.1038/s41598-025-00836-3

**Published:** 2025-05-13

**Authors:** Zhandong Pan, Xuemei Cai, Yongming Bo, Changsheng Guan, Liqun Cai, Fasih Ullah Haider, Xuchun Li, Haixia Yu

**Affiliations:** 1 College of Forestry, Gansu Agricultural University, Lanzhou, 730070 China; 2 State Key Laboratory of Aridland Crop Science, Gansu Agricultural University, Lanzhou, 730070 China; 3 Dingxi Institute of Soil and Water Conservation Science Research, Dingxi, 743000 China; 4 College of Resources and Environment Sciences, Gansu Agricultural University, Lanzhou, 730070 China

Correction to: *Scientific Reports* 10.1038/s41598-024-82300-2, published online 30 December 2024

The original version of this Article contained an error in the spelling of the author Zhandong Pan, which was incorrectly given as Zhandogn Pan.

The original Article has been corrected.

